# Diagnosis-Driven Targeted Therapy in Acute Myeloid Leukemia: Clinical Integration of Tyrosine Kinase, BCL-2, and CD33-Directed Strategies with Midostaurin, Venetoclax, and Gemtuzumab Ozogamicin

**DOI:** 10.3390/jcm15134886

**Published:** 2026-06-23

**Authors:** Piotr Kawczak, Katarzyna Kawczak, Tomasz Bączek

**Affiliations:** 1 Department of Pharmaceutical Chemistry, Faculty of Pharmacy, Medical University of Gdańsk, 80-416 Gdansk, Poland; tomasz.baczek@gumed.edu.pl; 2 PwC, Aleja Grunwaldzka 472C, 80-309 Gdansk, Poland; 3 Department of Nursing and Medical Rescue, Institute of Health Sciences, Pomeranian University in Słupsk, 76-200 Slupsk, Poland

**Keywords:** acute myeloid leukemia, midostaurin, venetoclax, gemtuzumab ozogamicin, FLT3 mutation, BCL-2 inhibitor, antibody–drug conjugate, molecular diagnosis, targeted therapy, personalized medicine

## Abstract

Acute myeloid leukemia (AML) is a biologically heterogeneous malignancy in which therapeutic decision-making is increasingly guided by molecular and immunophenotypic diagnostics. Advances in genomic profiling and risk stratification have enabled the integration of targeted agents into frontline and relapsed/refractory treatment strategies. Among these, midostaurin, venetoclax, and gemtuzumab ozogamicin represent paradigm-shifting therapies whose clinical benefit depends on accurate and timely diagnosis. This review examines the diagnostic frameworks that inform the use of these agents and discusses their incorporation into contemporary AML management. Midostaurin has demonstrated improved outcomes in patients with FLT3-mutated AML when combined with intensive chemotherapy, underscoring the importance of early molecular testing. Venetoclax, a BCL-2 inhibitor, has expanded therapeutic options for older or unfit patients when used with hypomethylating agents or low-dose cytarabine, with emerging evidence linking response to cytogenetic and molecular features. Gemtuzumab ozogamicin, an anti-CD33 antibody–drug conjugate, illustrates the clinical relevance of immunophenotypic assessment and risk-adapted dosing strategies. We highlight current evidence supporting diagnosis-driven therapy selection, practical considerations for clinical implementation, and ongoing challenges, including resistance mechanisms and optimal sequencing. Integrating precise diagnostic tools with targeted therapies represents a critical step toward personalized AML care and improved patient outcomes.

## 1. Introduction

AML is a biologically diverse and clinically aggressive hematologic malignancy characterized by the clonal expansion of immature myeloid precursors, resulting in ineffective hematopoiesis and rapid disease progression if left untreated [1,2,3,4,5,6]. Although AML accounts for only approximately 1% of all newly diagnosed cancers worldwide and has an estimated annual incidence of 3–5 cases per 100,000 persons, it remains one of the most lethal hematologic malignancies and a leading cause of leukemia-related mortality, particularly among older adults, in whom the median age at diagnosis exceeds 65 years [7,8,9,10,11,12]. The substantial mortality associated with AML reflects its aggressive clinical course, marked molecular and genetic heterogeneity, advanced age at presentation, and high rates of treatment resistance and disease relapse, resulting in a 5-year overall survival rate below 35% in most adult populations [3,4,5,6,7]. Consequently, despite significant therapeutic advances achieved over the past decade, AML continues to represent a major unmet medical need [9,10,11,12]. The clinical course of AML is highly variable, reflecting its underlying molecular heterogeneity, which encompasses a wide spectrum of cytogenetic abnormalities, gene mutations, and epigenetic alterations that collectively influence disease biology, treatment response, and prognosis [13,14,15,16,17,18].

For several decades, the therapeutic paradigm for AML remained largely unchanged, centered on intensive induction chemotherapy with anthracycline- and cytarabine-based regimens, followed by consolidation with additional chemotherapy or allogeneic hematopoietic stem cell transplantation (allo-HSCT) in eligible patients [19,20,21,22,23,24]. While these approaches achieved meaningful remission rates in younger, fit individuals, their efficacy in older or medically unfit populations was limited, and relapse remained the principal cause of treatment failure across all age groups [25,26,27,28,29]. Importantly, this “one-size-fits-all” strategy did not account for the biological diversity of AML, leading to significant variability in outcomes even among patients receiving identical treatment regimens [30,31,32,33].

The past decade has witnessed a profound transformation in the understanding and management of AML, driven by advances in genomic technologies such as next-generation sequencing (NGS), which have enabled comprehensive characterization of the mutational landscape of the disease [34,35,36,37,38]. Recurrent alterations in genes such as FLT3, NPM1, IDH1/2, DNMT3A, TP53, and others have been identified as key drivers of leukemogenesis, offering both prognostic insights and actionable therapeutic targets [39,40,41]. These discoveries have catalyzed a paradigm shift toward precision medicine, in which diagnostic evaluation extends beyond morphology and immunophenotyping to include integrated molecular profiling that directly informs therapeutic decision-making [42,43,44].

Contemporary classification systems, including the World Health Organization (WHO) and European LeukemiaNet (ELN) frameworks, now incorporate genetic abnormalities into disease definition and risk stratification, underscoring the central role of diagnosis in guiding treatment selection [45,46,47]. This evolution reflects a broader transition in oncology from empiric therapy toward diagnosis-driven strategies, wherein treatment is tailored to the specific biological features of an individual patient’s disease. In AML, this approach has led to the development and regulatory approval of multiple targeted therapies that exploit defined molecular vulnerabilities [48,49,50].

In this review, we focus on midostaurin, venetoclax, and gemtuzumab ozogamicin (GO), three agents that exemplify distinct yet complementary therapeutic strategies and collectively reflect the evolution of AML management toward precision medicine. Midostaurin targets aberrant FLT3 signaling, venetoclax restores apoptotic susceptibility through selective BCL-2 inhibition, and GO delivers targeted cytotoxic therapy via CD33-directed antibody–drug conjugation. Together, these agents demonstrate how molecular and immunophenotypic characteristics can be leveraged to guide treatment selection and optimize clinical outcomes.

Among these therapies, midostaurin, a multikinase inhibitor with potent activity against FLT3, was one of the first targeted agents to demonstrate a significant overall survival (OS) benefit when incorporated into standard induction chemotherapy for patients with FLT3-mutated AML, as shown in the landmark RATIFY trial [51,52,53]. This pivotal study established proof of concept for the integration of molecularly targeted therapies into frontline treatment regimens, demonstrating that biomarker-guided therapeutic interventions can improve outcomes without compromising the efficacy of conventional chemotherapy.

Venetoclax, a highly selective inhibitor of the anti-apoptotic protein BCL-2, has transformed the treatment paradigm for older adults and patients deemed ineligible for intensive chemotherapy, particularly when combined with hypomethylating agents (HMAs) such as azacitidine or decitabine [54,55,56]. By restoring apoptotic signaling in leukemic cells, venetoclax-based regimens have produced high response rates and prolonged survival in populations historically associated with poor therapeutic outcomes, thereby addressing a major unmet clinical need [57,58,59]. Furthermore, accumulating evidence indicates that specific molecular subgroups, including AML harboring NPM1 or IDH mutations, may exhibit enhanced sensitivity to venetoclax-based therapy, underscoring the growing importance of biomarker-driven patient stratification [60].

GO, a CD33-directed antibody–drug conjugate, represents one of the earliest targeted therapies developed for AML and serves as an instructive example of the refinement of precision oncology strategies over time [61,62,63]. Although initially withdrawn from clinical use because of safety concerns, particularly hepatic veno-occlusive disease, GO was subsequently reintroduced using lower, fractionated dosing schedules following evidence of improved tolerability and clinical efficacy in appropriately selected patient populations, particularly adults with newly diagnosed CD33-positive de novo AML who were eligible for intensive induction chemotherapy [64]. Subsequent studies demonstrated that the addition of GO to induction chemotherapy can improve survival outcomes in patients with favorable- or intermediate-risk cytogenetic profiles, highlighting the value of risk-adapted treatment approaches in AML [65].

Despite these advances, several challenges and areas of ongoing debate remain. One major controversy concerns the optimal integration of targeted agents into existing treatment algorithms, particularly in the context of combination versus sequential strategies and the role of maintenance therapy [66]. Additionally, the increasing complexity of molecular diagnostics raises questions regarding the timing, scope, and clinical interpretation of genomic testing, as well as issues related to accessibility and cost-effectiveness in routine practice [67]. The emergence of clonal evolution and therapeutic resistance further complicates treatment decision-making, underscoring the need for dynamic monitoring approaches such as measurable residual disease (MRD), formerly commonly referred to as minimal residual disease assessment and serial molecular profiling [68,69].

Another area of active investigation involves the identification of predictive biomarkers beyond single-gene mutations, including gene expression signatures, epigenetic profiles, and functional dependencies that may refine patient stratification and guide therapeutic selection [70]. Moreover, while targeted therapies have improved outcomes in specific subsets of AML, their long-term impact on survival, quality of life, and curative potential remains to be fully elucidated, particularly in comparison with established modalities such as HSCT [71,72]. These considerations highlight the evolving and, at times, controversial nature of precision medicine in AML, where rapid scientific progress must be balanced with careful clinical validation.

In this context, the present narrative review aims to provide a comprehensive and clinically oriented overview of diagnosis-driven targeted therapy in AML, focusing on the integration of midostaurin, venetoclax, and GO into contemporary management strategies. We examine the biological rationale underlying their use, summarize key clinical trial data supporting their efficacy, and discuss practical considerations for their implementation in routine clinical practice. Particular emphasis is placed on the role of diagnostic modalities—including molecular profiling, immunophenotyping, and MRD assessment—in guiding treatment selection and monitoring response.

The principal conclusion of this review is that the integration of precise diagnostic tools with targeted therapeutic interventions has fundamentally transformed the management of AML, shifting the field toward a more individualized and biologically informed approach. However, the successful implementation of this paradigm requires not only continued advances in drug development but also the optimization of diagnostic workflows, interdisciplinary collaboration, and equitable access to molecular testing. Ultimately, the convergence of diagnostics and therapeutics holds the promise of improving outcomes across diverse patient populations and advancing the broader goals of precision oncology in hematologic malignancies.

This article was conducted as a narrative review using a structured literature-search approach. Relevant English-language publications were identified through searches of PubMed/MEDLINE, Scopus, and Web of Science databases covering the period from 2006 to May 2026. The selected timeframe was intended to capture both the early translational and clinical investigations that preceded the regulatory development of targeted therapies in AML, as well as contemporary studies defining current standards of care. Search terms included combinations of “acute myeloid leukemia,” “midostaurin,” “venetoclax,” “gemtuzumab ozogamicin,” “FLT3,” “BCL-2,” “CD33,” “targeted therapy,” and “combination therapy.” Additional relevant references were identified through manual screening of bibliographies from eligible publications. Eligible studies included phase II and III prospective clinical trials, randomized controlled trials, registration-directed studies, long-term follow-up analyses, meta-analyses, and clinically informative prospective cohort studies evaluating the efficacy and safety of midostaurin, venetoclax, and gemtuzumab ozogamicin. Priority was given to pivotal trials supporting regulatory approvals and major international guideline documents, while large real-world studies and retrospective analyses were included when they provided meaningful insights into treatment outcomes, safety profiles, resistance mechanisms, patient selection, or therapeutic sequencing. Given the narrative nature of this review, formal PRISMA methodology, systematic risk-of-bias assessment, and quantitative evidence synthesis were not performed.

The schematic figures included in this manuscript were created by the authors using graphical software that may incorporate artificial intelligence–assisted functionalities to support visualization. All scientific content, interpretation, and final figure design were critically reviewed and approved by the authors, who take full responsibility for the accuracy and originality of the material.

**Figure 1:**
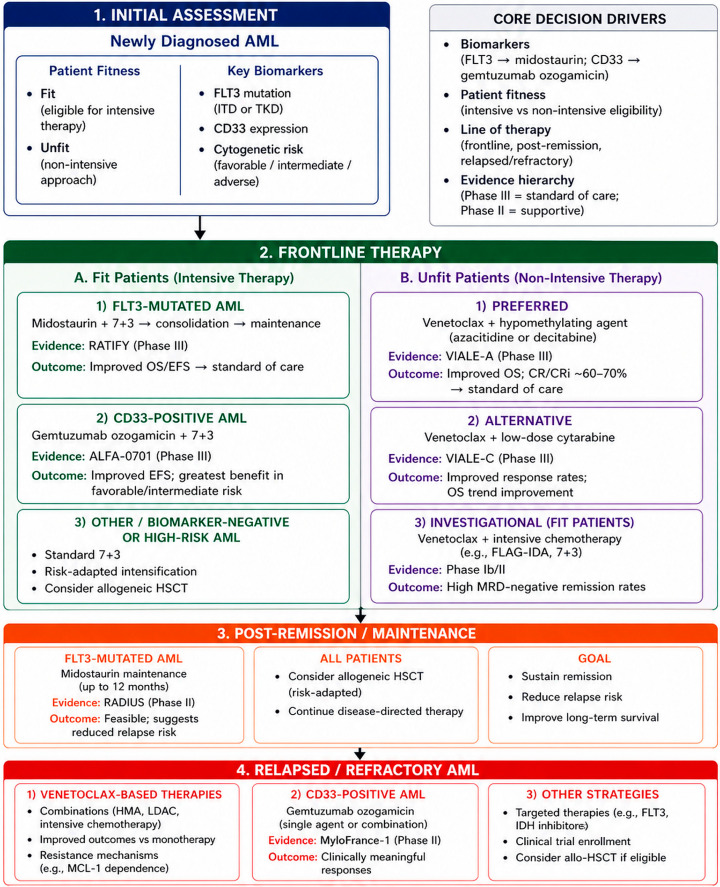
Schematic comparison overview of treatment selection in acute myeloid leukemia, integrating pivotal clinical trial data by biomarker status and line of therapy with the use of midostaurin, venetoclax, and gemtuzumab ozogamicin. The scheme represents an original interpretative synthesis of the currently available evidence, developed in accordance with key NCCN and ESMO principles, including biomarker-driven therapeutic selection and treatment sequencing strategies. It constitutes an original graphical representation prepared by the authors and does not reproduce, duplicate, or replicate any specific published guideline, figure, or external source. All abbreviations employed are defined in the text in the Abbreviations section.

Figure 1 provides a schematic overview of diagnosis-driven treatment selection in AML, integrating key clinical trial evidence according to molecular profile, cytogenetic risk, and line of therapy.

## 2. Midostaurin

**Figure 2:**
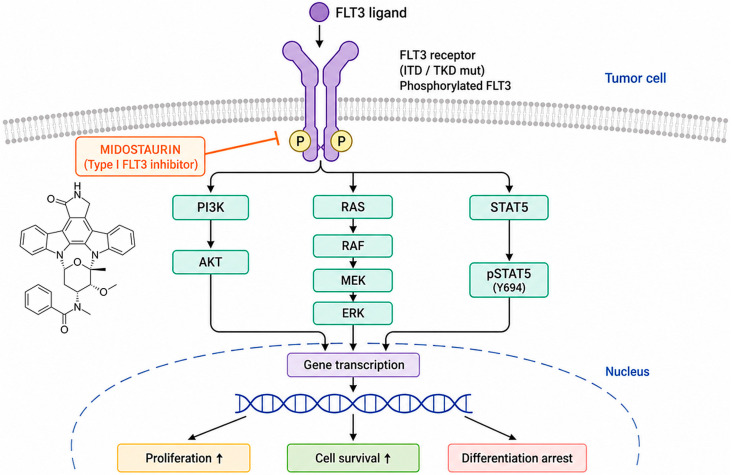
Midostaurin mechanism of action. Original schematic illustration created by the authors based on the mechanism described in [89]. FLT3 activation—most commonly through internal tandem duplication (ITD) or tyrosine kinase domain (TKD) mutations—triggers downstream signaling via PI3K/AKT, RAS/ERK, and STAT5 pathways, promoting leukemic cell proliferation, survival, and impaired differentiation. Midostaurin, a type I FLT3 inhibitor, binds to the active conformation of the receptor and suppresses its phosphorylation, thereby attenuating downstream signaling and reducing leukemic cell growth. All abbreviations employed are defined in the text in the Abbreviations section.

Midostaurin is a first-generation, orally administered, multi-targeted tyrosine kinase inhibitor that inhibits FLT3 as well as KIT, PDGFR, VEGFR, and other kinases involved in leukemic proliferation, survival, and microenvironmental signaling [73,74,75,76]. In AML, its clinical relevance is primarily linked to FLT3-mutated disease, particularly FLT3-ITD and FLT3-TKD mutations, which occur in a substantial proportion of patients and are associated with increased relapse risk and adverse prognosis, especially in cases with high FLT3-ITD allelic burden or coexisting high-risk molecular features [77,78,79,80]. By inhibiting constitutively activated FLT3 signaling, midostaurin suppresses downstream PI3K/AKT, RAS/MAPK, and STAT5 pathways, thereby reducing blast proliferation and promoting apoptosis [81,82,83,84,85,86]. However, because AML is biologically heterogeneous and frequently driven by cooperating mutations, FLT3 inhibition alone is rarely sufficient to produce durable disease control, which explains why midostaurin has achieved its greatest clinical impact when combined with intensive chemotherapy rather than as monotherapy [78,87,88]. Figure 2 presents the mechanism of action of midostaurin.

Early clinical experience with midostaurin in relapsed or refractory AML and high-risk myelodysplastic syndromes demonstrated clear biological activity but limited durability of response [90]. In the phase IIb monotherapy study by Fischer et al., midostaurin reduced peripheral and bone marrow blasts in a subset of patients, including those with FLT3-mutated disease, although complete remissions (CRs) were uncommon and responses were generally transient [90]. These findings were important not because they established midostaurin as an effective single agent, but because they validated FLT3 as a therapeutically actionable target and provided a rationale for combination-based strategies.

Consistent with this, a phase I/II study combining midostaurin with azacitidine demonstrated feasibility and modest activity in AML and high-risk MDS, although responses remained variable and not durable [91]. The subsequent phase Ib study by Stone et al. demonstrated that midostaurin could be safely integrated with standard daunorubicin and cytarabine induction followed by consolidation therapy in younger adults with newly diagnosed AML, establishing a feasible dosing framework that directly informed the design of the pivotal RATIFY trial [92]. Together, these studies highlight a central principle in AML therapeutics: targeted agents such as FLT3 inhibitors achieve clinically meaningful benefit primarily when incorporated into established treatment backbones, a concept further reinforced by later combination strategies, including venetoclax-based regimens in non-intensive settings.

The pivotal RATIFY/CALGB 10603 trial (*n* = 717) transformed the management of newly diagnosed FLT3-mutated AML [93]. In this phase III, randomized, double-blind, placebo-controlled study, adults aged 18–59 years with newly diagnosed FLT3-ITD or FLT3-TKD AML received standard daunorubicin/cytarabine induction and high-dose cytarabine consolidation with either midostaurin or placebo, followed by maintenance therapy. The addition of midostaurin significantly improved OS and event-free survival compared with placebo, establishing midostaurin plus intensive chemotherapy as a frontline standard of care in fit patients with FLT3-mutated AML [93]. Importantly, benefit was observed across FLT3 mutation subgroups, including both ITD and TKD mutations, supporting broad use within molecularly eligible patients [93,94,95].

From a critical perspective, however, RATIFY enrolled only younger, intensive-therapy-eligible patients, limiting direct extrapolation to older or frailer populations. Moreover, the contribution of maintenance therapy and the influence of allo-HSCT complicate interpretation of the independent magnitude of benefit attributable to midostaurin during induction and consolidation [93,94,96,97]. Nevertheless, the trial remains clinically decisive because it demonstrated that early FLT3-directed therapy can improve survival when introduced at diagnosis rather than reserved for relapse.

The AMLSG 16-10 trial (*n* = 440) further supported the integration of midostaurin into frontline treatment for FLT3-ITD AML across a broader age range [98]. This phase II, single-arm study evaluated midostaurin with intensive chemotherapy followed by continuation therapy in adults aged 18–70 years, including patients who proceeded to allo-HSCT. Compared with historical controls, outcomes appeared improved, supporting the feasibility of midostaurin in both younger and older fit patients and across different post-remission strategies [98]. However, because AMLSG 16-10 was not randomized, its conclusions are inherently limited by comparisons with historical cohorts, potential selection bias, and changes in supportive care over time. Even so, the study is clinically useful because it reflects treatment patterns closer to real-world AML practice than RATIFY and reinforces the concept that midostaurin can be incorporated around transplant-based strategies.

Additional evidence comes from the global phase 3b A2408 study (*n* = 301), an open-label, multicenter, single-arm trial evaluating midostaurin in combination with standard induction and consolidation chemotherapy followed by maintenance therapy in adults aged 18–70 years with newly diagnosed FLT3-mutated AML [99]. This study confirmed the safety and effectiveness of midostaurin in a broader, more real-world-like population, including older fit patients eligible for intensive chemotherapy [99]. Although the absence of a control arm prevents definitive efficacy comparisons, A2408 is important because it addresses a practical limitation of RATIFY: many patients encountered in routine AML care fall outside the strict age and eligibility criteria of pivotal randomized trials. Together, RATIFY, AMLSG 16-10, and A2408 support the use of midostaurin as part of frontline intensive therapy in molecularly selected patients, while also emphasizing that patient fitness, transplant eligibility, and treatment goals remain central to therapeutic decision-making [93,98,99].

The post-transplant role of midostaurin has been explored most directly in the RA-DIUS trial (*n* = 60) [100]. This phase II, randomized, open-label study evaluated standard of care with or without midostaurin maintenance in patients with FLT3-ITD-positive AML in first CR after allo-HSCT. The study demonstrated the feasibility of post-transplant FLT3 inhibition and suggested a reduction in relapse risk, with estimated 18-month relapse-free survival rates of 89% in the midostaurin arm versus 76% in the standard-of-care arm. However, the study was not powered to demonstrate statistically significant differences in relapse-free or overall survival [100]. Therefore, RADIUS should be interpreted as supportive rather than conclusive evidence for post-transplant midostaurin maintenance. Its clinical relevance lies in showing that FLT3 inhibition after transplant is biologically and operationally plausible, particularly in patients at high risk of relapse. However, in contemporary practice, the post-transplant maintenance space is increasingly influenced by more selective FLT3 inhibitors, and direct comparative evidence remains limited [101,102,103,104].

Beyond FLT3 inhibition, midostaurin exhibits activity against several class III receptor tyrosine kinases and signaling proteins, including KIT, PDGFR, VEGFR2, and protein kinase C. KIT inhibition may be particularly relevant in core-binding factor (CBF) AML, especially in patients with t(8;21) AML, where activating KIT mutations occur frequently and have been associated with increased relapse risk and inferior clinical outcomes. Preclinical studies have demonstrated that midostaurin effectively inhibits KIT-dependent signaling pathways, thereby reducing leukemic cell proliferation and survival. Clinical and translational studies have further highlighted KIT as an important pharmacologic target of midostaurin and suggested that its multikinase activity contributes substantially to its therapeutic efficacy in selected AML populations and other KIT-driven hematologic malignancies [99,105,106]. Additional evidence supporting the clinical relevance of KIT inhibition has been derived from studies in advanced systemic mastocytosis, where midostaurin demonstrated meaningful activity against KIT-driven disease, further supporting its broad kinase-targeting profile [107,108].

This broader kinase inhibitory profile has prompted investigation of midostaurin in molecularly defined AML subsets beyond classical FLT3-mutated disease. The SAL MIDOKIT trial (*n* = 77), a phase II, single-arm study, evaluated midostaurin in combination with intensive chemotherapy in patients with t(8;21) AML harboring KIT and/or FLT3 mutations [99]. The study reported encouraging responses in this high-risk molecular subgroup, providing clinical evidence that targeting KIT-driven signaling may improve outcomes and suggesting that midostaurin’s activity against KIT may be clinically relevant beyond its established role as a FLT3 inhibitor [99]. Consequently, the clinical benefits observed with midostaurin may not be exclusively attributable to FLT3 inhibition but may also reflect broader modulation of oncogenic kinase networks involved in leukemogenesis and disease persistence [99,105,106].

However, these findings should be interpreted cautiously because of the relatively small sample size, single-arm design, and biological heterogeneity within CBF-AML. Therefore, although the SAL MIDOKIT trial expands the conceptual role of midostaurin beyond FLT3-mutated AML and supports further exploration of KIT-directed therapeutic strategies, it does not redefine the drug’s principal clinical indication, which remains FLT3-mutated AML treated with intensive chemotherapy [99].

The adverse-effect profile of midostaurin reflects both its multikinase activity and its use alongside intensive chemotherapy. Common toxicities include nausea, vomiting, diarrhea, mucositis, rash, febrile neutropenia, anemia, thrombocytopenia, and infections, many of which overlap with expected complications of AML induction therapy [99,109,110,111]. Less frequent but clinically relevant events include QT interval prolongation and pulmonary toxicity, including interstitial lung disease or pneumonitis, which require careful monitoring and prompt evaluation of respiratory symptoms [109,112]. In RATIFY, the addition of midostaurin did not substantially increase treatment-related mortality, supporting its safety in appropriately selected patients [93]. Nevertheless, tolerability can be more challenging in older adults, and drug–drug interactions are clinically important because midostaurin is metabolized by CYP3A4, making concomitant azole antifungals and other CYP3A4 inhibitors relevant during AML induction [113,114,115].

Clinically, midostaurin is best understood as a foundational targeted therapy that established FLT3 inhibition as a standard component of diagnosis-driven AML management. Its strengths include randomized evidence of survival benefit, activity across FLT3-ITD and FLT3-TKD mutations, feasibility with intensive chemotherapy, and a manageable safety profile [94,99,116,117]. Its limitations include modest single-agent activity, lack of direct comparison with newer FLT3 inhibitors in the frontline setting, uncertain independent contribution of maintenance therapy, and reduced clarity in older or non–intensive-therapy-eligible patients [90,93,101]. The emergence of more selective FLT3 inhibitors, including gilteritinib, quizartinib, and crenolanib, has refined the therapeutic landscape and raised questions about optimal sequencing, post-remission strategies, and resistance management [118,119,120,121]. Among these agents, quizartinib and gilteritinib are the most clinically relevant comparators because both have demonstrated survival benefits in phase III clinical trials and have been incorporated into contemporary treatment algorithms. Quizartinib is a highly selective type II FLT3 inhibitor with potent activity against FLT3-ITD mutations and was shown in the QuANTUM-First trial to improve overall survival when combined with intensive induction and consolidation chemotherapy in newly diagnosed FLT3-ITD-positive AML [122]. In contrast, gilteritinib is a type I FLT3 inhibitor active against both FLT3-ITD and FLT3-TKD mutations and has become a standard of care in relapsed or refractory FLT3-mutated AML following the ADMIRAL trial, which demonstrated superior survival compared with salvage chemotherapy [101].

From a mechanistic perspective, the broader kinase inhibition profile of midostaurin may contribute to its activity in selected molecular subgroups, including KIT-mutated CBF AML. However, its relatively limited FLT3 specificity may also result in less sustained target inhibition compared with newer agents. By contrast, quizartinib achieves more potent FLT3-ITD suppression but lacks significant activity against FLT3-TKD mutations, whereas gilteritinib maintains activity against both mutation classes and can overcome some mechanisms of resistance associated with earlier FLT3 inhibitors [101,118,119,120,121,122]. Consequently, these newer agents may provide deeper and more durable FLT3 blockade; however, direct comparisons with midostaurin remain challenging because available evidence derives from different patient populations, treatment settings, and clinical trial designs. To date, no randomized head-to-head studies have directly compared midostaurin with quizartinib or gilteritinib in newly diagnosed AML [93,101,122].

However, until direct comparative frontline data are available, midostaurin remains a key standard for fit patients with newly diagnosed FLT3-mutated AML receiving intensive induction therapy. Its development also illustrates a broader principle relevant to this review: targeted therapy in AML is most effective when integrated with diagnostic precision, risk-adapted consolidation, transplant planning, and careful toxicity management rather than used as an isolated molecular intervention [123,124,125,126].

Table 1 presents treatment-emergent adverse events and management strategies associated with midostaurin, while Table 2 presents major pivotal clinical trials and selected emerging studies evaluating midostaurin in AML.

## 3. Venetoclax

Venetoclax is a first-in-class, orally bioavailable, selective inhibitor of the anti-apoptotic protein B-cell lymphoma 2 (BCL-2), representing a paradigm shift in the therapeutic targeting of apoptotic dysregulation in AML [128,129,130,131]. Unlike kinase inhibitors such as midostaurin, which act upstream at the level of proliferative signaling, venetoclax directly engages the intrinsic apoptotic machinery by mimicking BH3-only proteins and displacing pro-apoptotic factors, such as BIM, from BCL-2, thereby restoring mitochondrial outer membrane permeabilization and caspase activation [132,133,134,135,136,137]. This mechanism is particularly relevant in AML, where leukemic stem and progenitor cells frequently exhibit BCL-2 dependence, especially in less proliferative, metabolically quiescent clones [138,139,140,141].

Beyond its canonical pro-apoptotic effects, venetoclax exerts profound effects on leukemic cellular metabolism. Increasing evidence indicates that AML cells, particularly leukemia stem cells (LSCs), are highly dependent on mitochondrial oxidative phosphorylation (OXPHOS) rather than glycolysis for energy production and long-term survival [138,139,142,143]. BCL-2 contributes not only to apoptotic resistance but also to the maintenance of mitochondrial integrity and respiratory function. By inhibiting BCL-2, venetoclax disrupts mitochondrial homeostasis, reduces mitochondrial membrane potential, suppresses OXPHOS activity, and diminishes ATP production, thereby impairing the metabolic fitness of LSCs that are believed to contribute to disease persistence and relapse [138,139,142,143]. The metabolic consequences of venetoclax appear particularly important when the drug is combined with hypomethylating agents such as azacitidine. Preclinical and translational studies have demonstrated that azacitidine further suppresses amino acid uptake and metabolism, limiting substrates required for mitochondrial respiration and creating a dual metabolic blockade that selectively targets OXPHOS-dependent LSCs while relatively sparing normal hematopoietic stem cells [138,142]. Furthermore, emerging evidence suggests that metabolic adaptations involving fatty acid oxidation, amino acid metabolism, mitochondrial remodeling, and the emergence of monocytic subclones may contribute to acquired venetoclax resistance, highlighting cellular energetics as an important therapeutic vulnerability in AML [139].

Importantly, apoptotic dependency in AML is heterogeneous and can shift toward alternative anti-apoptotic proteins such as MCL-1 or BCL-XL, providing a biological basis for both intrinsic and acquired resistance to venetoclax [144,145,146,147,148]. Consequently, the clinical efficacy of venetoclax is increasingly recognized as arising not only from induction of apoptosis through BCL-2 inhibition but also from disruption of mitochondrial energy metabolism essential for leukemic cell survival and stemness [138,139,142,143].

**Figure 3:**
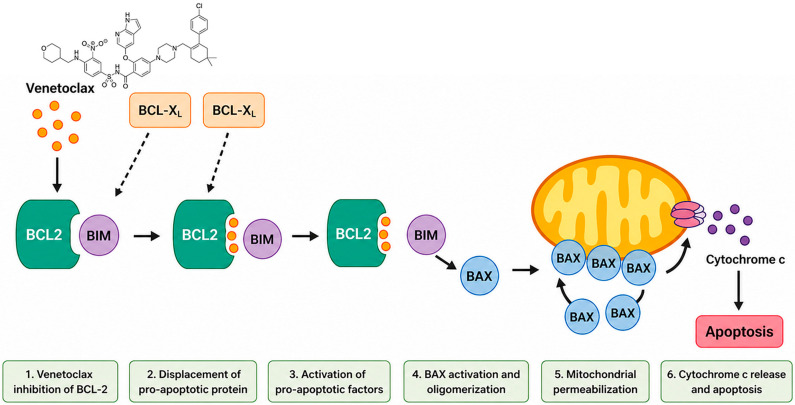
Venetoclax mechanism of action. Original schematic illustration created by the authors based on the mechanism described in [149]. Venetoclax selectively inhibits the anti-apoptotic protein BCL-2. Under physiological conditions, BCL-2 sequesters pro-apoptotic proteins such as BIM, thereby preventing activation of BAX and maintaining mitochondrial integrity. In the schematic, BCL-X_L_ is also shown as an additional anti-apoptotic protein that can bind pro-apoptotic factors and support cell survival, but it is not effectively inhibited by venetoclax. Upon venetoclax binding to BCL-2, BIM is displaced and released, enabling activation of the pro-apoptotic effector BAX. Activated BAX translocates to the mitochondrial outer membrane, where it oligomerizes and induces membrane permeabilization. This leads to the release of cytochrome c into the cytosol and subsequent activation of the apoptotic cascade, ultimately resulting in programmed cell death. All abbreviations employed are defined in the text in the Abbreviations section.

Figure 3 depicts the mechanism of action of venetoclax.

Initial clinical evaluation of venetoclax monotherapy in relapsed or refractory AML demonstrated clear biological activity but limited clinical durability, with modest response rates and short-lived remissions [150]. In the phase II study by Konopleva et al., single-agent venetoclax demonstrated modest clinical activity in relapsed or refractory AML, achieving an overall response rate (ORR) of 19%, including CR and complete remission with incomplete hematologic recovery (CRi) in 6% of patients, with higher response rates observed among patients harboring IDH mutations [146]. Although venetoclax induced reductions in blast counts and occasional durable remissions, the magnitude of response was insufficient to support its use as monotherapy. These findings underscored a critical principle: although BCL-2 inhibition effectively primes leukemic cells for apoptosis, it often requires combination with agents that induce pro-apoptotic stress to achieve sustained responses. Mechanistically, HMAs and low-dose cytarabine (LDAC) enhance mitochondrial priming and increase dependence on BCL-2, thereby providing a rational basis for combination therapy [21,151,152,153].

This strategy was validated in early-phase trials that combined venetoclax with HMAs or LDAC in older or unfit patients with newly diagnosed AML. The phase Ib/II study by DiNardo et al. demonstrated high composite CR/CRi rates of approximately 60–70% with venetoclax plus azacitidine or decitabine, with rapid responses and acceptable toxicity [114]. Similarly, the phase Ib/II study by Wei et al. showed improved response rates with venetoclax plus LDAC compared with historical LDAC monotherapy, supporting further development of this combination [87]. These early trials were notable not only for their efficacy but also for the speed of response, with many patients achieving remission within the first treatment cycles, a feature that has important clinical implications for bridging to transplant or reducing transfusion dependence.

The pivotal phase III VIALE-A trial (*n* = 433) established venetoclax in combination with azacitidine as a standard of care for patients with newly diagnosed AML who are ineligible for intensive chemotherapy [27]. In this randomized, double-blind study, venetoclax plus azacitidine significantly improved OS compared with azacitidine alone, along with higher CR/CRi rates and more frequent MRD negativity. The survival benefit was clinically meaningful and consistent across multiple subgroups, including patients with adverse-risk cytogenetics, although outcomes remained inferior in certain molecular subsets such as TP53-mutated disease [27,154,155]. Importantly, VIALE-A demonstrated that effective disease control could be achieved without intensive chemotherapy, fundamentally altering the treatment paradigm for older and comorbid AML patients.

The VIALE-C trial (*n* = 211) evaluated venetoclax in combination with LDAC in a similar patient population [126]. While this study showed improved response rates and a trend toward improved OS compared with LDAC alone, the magnitude of benefit was less pronounced than in VIALE-A. This difference likely reflects both biological and clinical factors, including the relatively weaker cytotoxic and epigenetic effects of LDAC compared with HMAs, as well as differences in patient populations and trial design. From a clinical perspective, these findings have positioned venetoclax plus azacitidine as the preferred non-intensive regimen, with venetoclax plus LDAC reserved for selected patients in whom HMAs are not suitable [119,156,157,158].

The phase Ib/II M14-358 study (*n* = 145) established the clinical activity of venetoclax in combination with azacitidine or decitabine in older patients with newly diagnosed AML who were ineligible for intensive chemotherapy, demonstrating high remission rates, durable responses, and a manageable safety profile. These findings provided the foundation for the subsequent phase III VIALE-A trial and the incorporation of venetoclax-based hypomethylating agent regimens into standard clinical practice. In contrast, the pivotal data supporting venetoclax in combination with LDAC were derived primarily from the phase Ib/II M14-387 study (*n* = 82) and later confirmed in the phase III VIALE-C trial. More broadly, differences between VIALE-A and VIALE-C highlight an important consideration in AML trial interpretation: outcomes are influenced not only by the targeted agent but also by the backbone therapy, patient selection, and endpoint definitions. Consequently, cross-trial comparisons should be interpreted with caution [159].

In relapsed or refractory AML, venetoclax-based combinations have shown improved activity compared with monotherapy, but responses are generally less durable than in the frontline setting [160,161,162]. Resistance mechanisms are increasingly recognized and include upregulation of alternative anti-apoptotic proteins such as MCL-1, clonal evolution, and metabolic reprogramming [123,146,163,164]. These findings have stimulated interest in rational combination strategies, including pairing venetoclax with MCL-1 inhibitors, FLT3 inhibitors, or IDH inhibitors, although many of these approaches remain investigational.

Emerging studies have also explored the integration of venetoclax into intensive chemotherapy regimens for younger, fit patients. Early-phase trials combining venetoclax with FLAG-IDA or standard “7+3” induction have reported high rates of MRD-negative remission, suggesting that deep molecular responses may be achievable with this approach [120]. However, these regimens are associated with increased myelosuppression and infectious complications, and optimal dosing schedules remain under investigation. Importantly, the available evidence is derived primarily from single-arm, non-randomized studies involving highly selected patient populations, including younger individuals with favorable performance status who are eligible for intensive therapy. As a result, treatment outcomes may be influenced by selection bias, differences in baseline disease characteristics, and other confounding factors that cannot be adequately controlled in the absence of randomized comparators. Furthermore, the lack of control arms limits the ability to determine whether the favorable remission rates observed are attributable to venetoclax itself or to patient selection and intensive chemotherapy backbones. Consequently, the generalizability of these findings to broader AML populations remains uncertain. Thus, while venetoclax-intensified chemotherapy represents a promising therapeutic strategy, its precise clinical benefit and risk–benefit profile require confirmation in prospective randomized trials before routine adoption can be recommended.

The safety profile of venetoclax is characterized by predictable and mechanism-based toxicities. Myelosuppression, including prolonged neutropenia and thrombocytopenia, is the most significant adverse effect and frequently necessitates dose interruptions, cycle delays, growth factor support, and careful monitoring [27,165,166,167]. Infectious complications are also common, reflecting both disease-related immunosuppression and treatment-induced cytopenias [168,169]. Tumor lysis syndrome (TLS), although less common in AML than in chronic lymphocytic leukemia, remains a clinically relevant risk, particularly during treatment initiation, and requires appropriate prophylaxis and laboratory monitoring [28]. Gastrointestinal adverse events, including nausea and diarrhea, are generally mild and manageable.

The pharmacokinetics of venetoclax are strongly influenced by CYP3A4-mediated metabolism, creating substantial potential for clinically significant drug–drug interactions [170,171]. Consequently, co-administration of strong CYP3A4 inhibitors, including commonly used azole antifungal agents such as posaconazole, voriconazole, and itraconazole, requires substantial venetoclax dose reductions to minimize excessive drug exposure and treatment-related toxicity [170,171]. Conversely, strong CYP3A4 inducers, including rifampin, carbamazepine, phenytoin, and St. John’s wort, can markedly reduce venetoclax plasma concentrations and potentially compromise therapeutic efficacy [172]. For this reason, major venetoclax clinical trials, including VIALE-A and VIALE-C, incorporated protocol-defined dose modifications and monitoring strategies to account for clinically significant CYP3A4 drug interactions [56].

Beyond drug–drug interactions, emerging evidence suggests that interindividual variability in CYP3A4 activity may contribute to differences in venetoclax exposure, treatment response, and toxicity. Genetic polymorphisms affecting CYP3A4 expression or activity, as well as variability in hepatic function, concomitant medications, and inflammatory status, may alter venetoclax pharmacokinetics. Recent reviews have highlighted the potential relevance of pharmacogenetic and pharmacokinetic factors in optimizing venetoclax therapy, although routine CYP3A4 genotyping is not currently recommended in clinical practice [172,173]. Furthermore, population pharmacokinetic analyses and real-world therapeutic drug monitoring studies have demonstrated substantial variability in venetoclax exposure among AML patients, particularly in the setting of concomitant azole prophylaxis [173,174]. Higher systemic exposure has been associated with an increased risk of prolonged cytopenias, infectious complications, and other treatment-related toxicities, whereas inadequate exposure may reduce antileukemic activity [168]. These findings emphasize the importance of individualized dosing strategies, careful management of CYP3A4-mediated drug interactions, and ongoing evaluation of pharmacokinetic-guided approaches to optimize the balance between efficacy and safety in venetoclax-based AML therapy [172,173,174].

From a clinical perspective, venetoclax-based regimens have fundamentally reshaped the management of AML in patients unfit for intensive therapy, providing a highly effective, relatively well-tolerated alternative to conventional low-intensity approaches [27,119,130,157]. However, several limitations warrant consideration. First, responses, although frequent, are not uniformly durable, particularly in high-risk molecular subgroups such as TP53-mutated AML [154]. Second, prolonged cytopenias can complicate treatment delivery and increase the risk of infection, requiring careful dose optimization and supportive care. Third, most evidence is derived from trials in older or unfit patients, and the role of venetoclax in younger, fit populations remains less clearly defined [120].

Importantly, venetoclax exemplifies the transition toward biology-driven AML therapy, where treatment selection is increasingly informed by molecular features, patient fitness, and therapeutic goals. Its success has also highlighted the importance of targeting non-proliferative leukemic cell populations, including leukemic stem cells, which are often resistant to conventional chemotherapy [138,139,140,141]. Ongoing research is focused on refining combination strategies, identifying predictive biomarkers of response, and overcoming resistance mechanisms through rational therapeutic design, including approaches involving FLT3 co-targeting, modulation of resistance-associated pathways, MRD monitoring, and nuclear export inhibition [175,176,177,178,179,180,181,182,183]. In this evolving landscape, venetoclax occupies a central role as a backbone of non-intensive AML therapy, while its integration into broader treatment paradigms continues to be defined by emerging clinical evidence and mechanistic insights.

Table 3 shows TEAEs and management strategies associated with venetoclax, whereas Table 4 major pivotal clinical trials and selected emerging studies evaluating venetoclax in AML.

## 4. Gemtuzumab Ozogamicin

**Figure 4:**
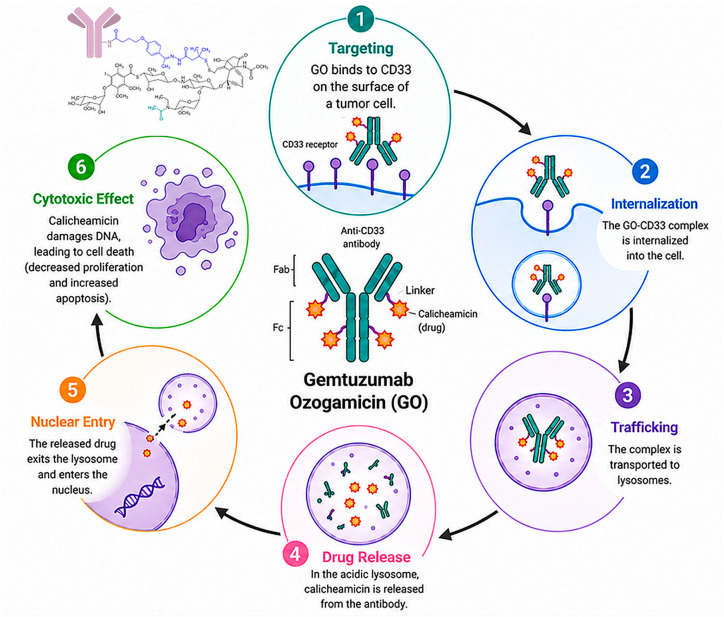
Gemtuzumab ozogamicin mechanism of action. Original schematic illustration created by the authors based on the mechanism described in [209]. CD33 is a sialic acid–dependent adhesion protein expressed on myeloid cells and present in ~90% of AML cases (>20% blasts), while absent on normal CD34+ stem cells and non-hematopoietic tissues. GO is a humanized anti-CD33 antibody conjugated to the cytotoxic agent calicheamicin. After binding CD33, the complex is internalized, calicheamicin is released in lysosomes, binds DNA, induces double-strand breaks, and triggers apoptosis. All abbreviations employed are defined in the text in the Abbreviations section.

GO is a CD33-directed antibody–drug conjugate composed of a humanized anti-CD33 monoclonal antibody linked to the cytotoxic calicheamicin derivative N-acetyl-γ-calicheamicin [191,192,193,194,195,196]. After binding to CD33 on leukemic blasts, the complex is internalized, the linker is hydrolyzed within lysosomes, and calicheamicin is released, inducing DNA double-strand breaks, mitochondrial injury, and apoptotic cell death [197,198,199,200]. Because CD33 is expressed on most AML blasts but not on normal hematopoietic stem cells, it became one of the earliest clinically exploited surface antigens in AML [200,201,202]. However, CD33 expression intensity, leukemic stem cell biology, cytogenetic risk, drug-efflux mechanisms, and treatment schedule all influence response, explaining why GO has shown marked benefit in some AML subsets but limited or harmful effects in others [203,204,205,206,207,208]. Figure 4 illustrates the mechanism of action of GO.

The first clinical development of GO focused on relapsed AML, where early-phase studies demonstrated meaningful responses in selected patients with CD33-positive disease [61,210,211]. These findings led to accelerated approval of GO for older patients with CD33-positive AML in first relapse, but subsequent experience revealed important safety concerns, especially hepatotoxicity and sinusoidal obstruction syndrome/veno-occlusive disease (SOS/VOD), particularly with higher single-dose schedules and in proximity to HSCT [212,213,214,215,216,217]. This early history is clinically important because it illustrates a central lesson in GO development: efficacy depends not only on antigen targeting but also on dose fractionation, patient selection, and integration with chemotherapy [214,218,219].

The MyloFrance-1 study (*n* = 57) was pivotal in redefining the dosing strategy of GO [18]. In this phase II trial, adults with CD33-positive AML in first relapse received fractionated single-agent GO, which produced clinically meaningful responses with a more acceptable safety profile than earlier higher-dose approaches [220]. Although the study was non-randomized and conducted in a selected relapsed population, it provided an important pharmacologic and clinical rationale for lower, fractionated dosing, which later became central to the renewed use of GO. This shift from high single-dose therapy to fractionated lower-dose administration helped reduce peak toxicity while preserving anti-leukemic activity [220,221,222].

The strongest evidence for GO in newly diagnosed adult AML comes from randomized frontline studies combining GO with intensive chemotherapy. In ALFA-0701 (*n* = 280), adults aged 50–70 years with newly diagnosed de novo AML were randomized to daunorubicin/cytarabine induction with or without fractionated GO [223]. GO significantly improved event-free survival and relapse-related outcomes, supporting its use in CD33-positive AML when administered in a fractionated low-dose schedule [223]. Final updates confirmed durable event-free survival benefit, although OS improvement was less consistent, partly reflecting post-remission therapy, salvage treatment, and competing mortality in older patients [64]. The clinical relevance of ALFA-0701 lies not only in the positive outcome but also in the schedule: fractionated GO improved the therapeutic index compared with earlier high-dose approaches [64,223].

Pediatric evidence also supports GO integration with chemotherapy. In the Children’s Oncology Group AAML0531 trial (*n* = 1022), children, adolescents, and young adults with newly diagnosed AML received standard chemotherapy with or without GO [224]. GO reduced relapse risk and improved event-free survival, although OS benefit was less pronounced [224]. This pattern—greater effect on relapse reduction than on OS—has been observed across several GO studies and likely reflects the impact of salvage therapies, transplantation, and competing toxicities. Nevertheless, in pediatric AML, where relapse prevention is a major determinant of long-term cure, the reduction in relapse risk is clinically meaningful [215,224,225].

Several large UK trials further clarified which patients derive the greatest benefit from GO. In NCRI AML16 (*n* = 1115), the addition of low-dose GO to induction chemotherapy improved survival in selected older patients with AML or high-risk myelodysplastic syndrome [226]. In MRC AML15 (*n* = 1113), younger adults receiving intensive therapy appeared to benefit most when they had favorable- or intermediate-risk cytogenetics, whereas patients with adverse-risk disease derived little benefit [227]. These results were reinforced by individual patient data meta-analysis, which showed that GO reduces relapse and improves survival mainly in favorable- and intermediate-risk AML, with limited benefit in adverse-risk disease [63]. This risk-adapted interpretation is essential: GO should not be viewed as a universally effective AML agent, but as a therapy whose value is greatest when disease biology is compatible with CD33-directed cytotoxic targeting [63,227].

Not all randomized trials were positive. SWOG S0106 (*n* = 637), which evaluated GO during induction and as post-consolidation therapy in younger adults with previously untreated de novo AML, failed to improve outcomes and was associated with increased early toxicity [228]. This negative study contributed to the temporary withdrawal of GO from the market and remains a critical cautionary example [228,229]. However, its findings should be interpreted in context: the trial used a different daunorubicin dose, GO schedule, and treatment design than later successful studies. Therefore, SWOG S0106 does not negate GO efficacy but emphasizes that dose intensity, scheduling, and patient selection determine whether CD33-directed therapy improves or worsens outcomes [228,229,230].

Additional randomized evidence from GOELAMS AML2006IR (*n* = 238) supported the role of GO in selected intermediate-risk AML, showing improved event-free survival but not OS when GO was added to intensive chemotherapy [231]. As with other studies, the discrepancy between event-free and OS highlights the complexity of AML outcome interpretation. Event-free survival may better capture relapse prevention, whereas OS is influenced by salvage therapy, transplant access, and treatment-related mortality [63,231]. Thus, GO’s most consistent clinical contribution is reduction in relapse risk, particularly in patients with favorable- or intermediate-risk disease [63,231].

The adverse-effect profile of GO is distinct from that of kinase inhibitors and BCL-2 inhibition because it combines myelosuppressive chemotherapy-like effects with antigen-directed hepatic toxicity. Common toxicities include prolonged cytopenias, infections, bleeding, infusion-related reactions, transaminase elevation, hyperbilirubinemia, and mucositis [64,223,228,232]. The most clinically significant toxicity is SOS/VOD, especially in patients receiving GO close to allo-HSCT, those with prior hepatic injury, or those exposed to higher cumulative doses [213,214,217,233]. Fractionated lower-dose regimens have reduced but not eliminated this risk [64,223,233]. Careful attention to hepatic function, transplant timing, cumulative exposure, and supportive care is therefore essential when incorporating GO into AML therapy.

In current clinical practice, GO is most relevant for patients with newly diagnosed CD33-positive AML receiving intensive chemotherapy, particularly those with favorable-risk CBF AML and selected intermediate-risk disease [234,235,236,237,238,239]. Its role is less clear in adverse-risk AML, where relapse biology is often driven by resistant clonal architecture rather than insufficient cytotoxic intensity alone [21,63]. GO may also be considered in selected relapsed CD33-positive AML, although contemporary relapse management increasingly includes molecularly targeted agents, venetoclax-based combinations, and transplant-directed strategies [21,240,241,242].

Overall, GO represents a mature example of antigen-directed therapy in AML: its clinical value is real but context-dependent, requiring integration of CD33 expression, cytogenetic risk, treatment intensity, transplant planning, and hepatic toxicity risk. Its development also demonstrates that targeted therapy in AML is not defined solely by molecular mutation status; surface-antigen targeting can meaningfully reduce relapse when appropriately matched to disease biology and treatment design [21,63,234,236].

Table 5 summarizes TEAEs and management strategies associated with gemtuzumab ozogamicin, whereas Table 6 summarizes major pivotal clinical trials and selected emerging studies evaluating GO in AML.

## 5. Future Directions in Precision Medicine and Clinical Management of Acute Myeloid Leukemia

The therapeutic landscape of acute myeloid leukemia (AML) is undergoing a profound transformation driven by advances in molecular biology, targeted therapy, and immuno-oncology, shifting clinical practice from empiric, largely cytotoxic approaches toward increasingly precise, diagnosis-driven strategies [245,246,247,248,249,250]. The integration of agents such as midostaurin, venetoclax, and GO into treatment algorithms exemplifies this transition, highlighting the clinical value of aligning therapy with specific molecular, cellular, and phenotypic disease features. However, despite these advances, AML remains characterized by substantial biological heterogeneity, clonal evolution, therapeutic resistance, and increasing economic complexity, underscoring that current progress represents an important but incomplete step toward fully personalized and sustainable care [159,235,248,251,252].

A central future direction in AML is the refinement of molecular stratification beyond single-driver mutations toward integrated genomic, transcriptomic, and epigenetic profiling. While current clinical decision-making relies heavily on recurrent alterations such as FLT3, IDH1/2, and TP53, emerging data indicate that co-mutation patterns, clonal architecture, and subclonal dynamics significantly influence treatment response and resistance [163,251,252,253]. For example, the efficacy of venetoclax-based regimens is modulated by molecular context, with favorable responses in IDH-mutated AML and more limited benefit in TP53-mutated disease, reflecting underlying differences in apoptotic dependency and genomic stability [139,163]. Similarly, the benefit of GO varies according to cytogenetic risk and CD33 expression levels, reinforcing the need for biomarker-informed patient selection rather than uniform application [204,254,255,256]. Future therapeutic strategies will likely incorporate composite biomarker models integrating genomic, immunophenotypic, and functional data to improve response prediction and treatment selection [205,257].

The increasing use of MRD assessment represents another critical evolution in AML management. High-sensitivity techniques, including multiparameter flow cytometry and NGS, enable detection of residual leukemic clones below morphologic thresholds and provide prognostic information that may inform post-remission strategies [129,258,259,260]. MRD-guided approaches offer the potential to refine decisions regarding consolidation therapy, transplant candidacy, maintenance treatment, and therapeutic sequencing. However, challenges remain, including assay standardization, interpretation across different molecular subtypes, and integration into clinical trial endpoints. Importantly, MRD negativity, while prognostically favorable, does not uniformly translate into cure, particularly in genetically complex disease, emphasizing the need for continued longitudinal monitoring and adaptive treatment strategies [259,260,261].

Resistance to targeted therapies remains a major limitation across currently available agents. Mechanisms of resistance are multifactorial and include secondary mutations, activation of parallel signaling pathways, epigenetic reprogramming, metabolic adaptation, and microenvironment-mediated survival signals [247,248,250,251]. Resistance to FLT3 inhibitors may arise through secondary kinase domain mutations or activation of alternative pathways such as RAS/MAPK, while resistance to venetoclax is frequently associated with upregulation of MCL-1 or BCL-XL [262,263]. These observations underscore the need for rational combination strategies designed to preempt or overcome resistance. Ongoing clinical trials are exploring combinations of targeted agents with complementary mechanisms, including FLT3 inhibitors with venetoclax, IDH inhibitors with HMAs, and triplet regimens incorporating multiple targeted pathways [264,265,266,267,268,269]. Although these approaches are promising, they also increase treatment complexity, toxicity burden, supportive care requirements, and overall healthcare resource utilization.

The role of immunotherapy in AML is an area of active investigation and represents a potential future pillar of treatment. Unlike lymphoid malignancies, AML has proven more challenging for immunotherapeutic approaches due to antigen heterogeneity, an immunosuppressive microenvironment, and shared antigen expression with normal hematopoietic cells [258,270,271,272]. Nevertheless, strategies including antibody–drug conjugates, bispecific T-cell engagers, immune checkpoint inhibitors, and CAR T-cell therapies are being developed and refined [156,273,274,275]. GO serves as a proof-of-concept for antigen-directed therapy in AML, although its clinical utility is limited by toxicity and variable efficacy across disease subtypes [63,266]. Next-generation immunotherapies aim to improve specificity, reduce off-target effects, and enhance immune activation, but their integration into standard care remains investigational and will depend on demonstrating durable benefit with acceptable safety profiles [87,263,276,277].

Another important direction is the expansion of targeted therapies into earlier lines of treatment and broader patient populations. While midostaurin and venetoclax have established roles in frontline settings for selected patient groups, their optimal sequencing, duration, and combination with other agents continue to evolve [189,278,279,280,281,282]. For example, the incorporation of venetoclax into intensive chemotherapy regimens for younger, fit patients has shown promising early results but raises concerns regarding cumulative myelosuppression, infectious complications, and prolonged treatment exposure [56,189]. Similarly, the role of maintenance therapy with targeted agents, particularly after allogeneic stem cell transplantation, remains an area of ongoing investigation, with the goal of reducing relapse while minimizing long-term toxicity [283,284]. These considerations highlight the need for carefully designed clinical trials addressing not only efficacy but also quality of life, long-term outcomes, and treatment sustainability.

Another important direction in AML therapy is the expansion of targeted treatments into earlier lines of care, broader patient populations, and increasingly refined molecular subgroups. While midostaurin and venetoclax have established roles in frontline settings for selected patient groups, their optimal sequencing, duration, and combination with other agents continue to evolve [189,278,279,280,281,282]. For example, the incorporation of venetoclax into intensive chemotherapy regimens for younger, fit patients has shown promising early results but raises concerns regarding cumulative myelosuppression, infectious complications, and prolonged treatment exposure [56,189]. Similarly, the role of maintenance therapy with targeted agents, particularly after allogeneic stem cell transplantation, remains an area of ongoing investigation, with the goal of reducing relapse while minimizing long-term toxicity [283,284].

A particularly promising therapeutic development is the emergence of menin inhibitors for genetically defined AML subsets, especially NPM1-mutated and KMT2A-rearranged disease. Menin functions as a critical epigenetic cofactor that interacts with KMT2A fusion proteins and contributes to the maintenance of oncogenic transcriptional programs, including aberrant expression of HOXA cluster genes and MEIS1, which play central roles in leukemic self-renewal, impaired differentiation, and disease persistence [285,286,287]. Pharmacologic disruption of the menin–KMT2A interaction downregulates these transcriptional programs, promotes myeloid differentiation, and suppresses leukemic proliferation. The strongest clinical evidence to date has been generated with revumenib and ziftomenib. In the phase I AUGMENT-101 trial, revumenib demonstrated meaningful clinical activity in heavily pretreated patients with KMT2A-rearranged or NPM1-mutated acute leukemias, producing complete remission or complete remission with partial hematologic recovery (CR/CRh) in approximately 30% of patients and enabling some individuals to proceed to allogeneic hematopoietic stem cell transplantation [285]. Similarly, early-phase studies of ziftomenib have shown encouraging response rates in relapsed or refractory NPM1-mutated AML, supporting further clinical development [286]. Importantly, menin inhibitors represent a mechanistically distinct therapeutic strategy that targets the transcriptional dependencies underlying leukemogenesis rather than conventional signaling pathways.

Ongoing studies are evaluating menin inhibitors in combination with intensive chemotherapy, hypomethylating agents, venetoclax, and FLT3 inhibitors to improve response depth and durability while preventing resistance [287]. If these approaches continue to demonstrate favorable efficacy and safety profiles, menin inhibition may become an important component of frontline treatment algorithms for molecularly defined AML subgroups. Together, these developments highlight the ongoing transition toward increasingly biomarker-driven and precision-based therapeutic strategies in AML. At the same time, they underscore the need for carefully designed clinical trials addressing not only efficacy, but also quality of life, long-term outcomes, treatment sustainability, and optimal integration of emerging targeted therapies into existing treatment paradigms [278,279,280,281,282,283,284].

From a broader clinical perspective, the implementation of precision medicine in AML must also account for cost-effectiveness, treatment accessibility, and healthcare system constraints. Targeted therapies are frequently associated with high acquisition costs, prolonged administration, intensive supportive care requirements, and expanded use of molecular diagnostics, including NGS and MRD monitoring [5,21,129]. Midostaurin has demonstrated both clinical and economic value in FLT3-mutated AML through improved survival and reduced relapse risk when combined with induction chemotherapy [93,116]. Venetoclax-based regimens have substantially improved outcomes in older or unfit AML patients, although treatment-associated cytopenias, infectious complications, and prolonged combination therapy may increase total healthcare costs [56,288,289]. Similarly, GO may provide cost utility in selected CD33-positive AML subsets by reducing relapse rates and improving remission durability, particularly within risk-adapted treatment strategies [223].

Future cost-effectiveness analyses will likely incorporate molecular risk stratification, treatment sequencing, quality-adjusted life years (QALYs), and real-world outcome data [4,10]. In parallel, biomarker-guided therapy and emerging artificial intelligence-assisted decision-support systems may improve therapeutic efficiency while optimizing healthcare resource allocation [248,290]. Importantly, disparities in access to comprehensive molecular diagnostics and high-cost targeted therapies remain major global challenges in AML care [21,283,291,292,293,294,295]. Moreover, the increasing complexity of treatment algorithms requires multidisciplinary expertise and specialized infrastructure, including rapid molecular testing, longitudinal monitoring, and advanced supportive care systems [288,296].

Ultimately, the future of AML management lies in the continued integration of molecular insights, targeted therapies, immunotherapeutic strategies, and adaptive treatment algorithms into a cohesive, patient-centered framework. While agents such as midostaurin, venetoclax, and GO have demonstrated that biologically informed therapy can improve outcomes, they also highlight persistent limitations related to resistance, toxicity, incomplete disease eradication, and financial sustainability. Progress will depend on the development of more precise biomarkers, rational combination regimens, improved MRD-guided approaches, equitable access to molecular diagnostics, and economically sustainable implementation of precision oncology strategies [5,10]. Achieving durable remission and cure in AML will ultimately require not only targeting dominant oncogenic drivers but also addressing the broader complexity of leukemic biology, including clonal heterogeneity, leukemic stem cell persistence, microenvironmental interactions, and healthcare system realities [245,246,247,248,263,264].

**Figure 5:**
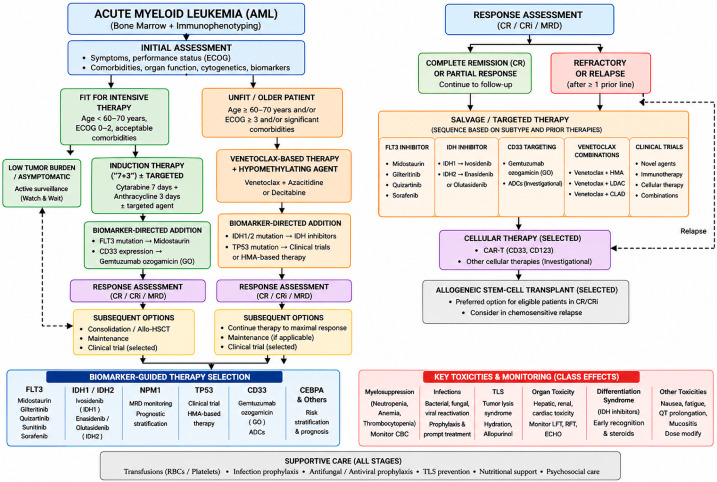
Therapeutic pathways and treatment algorithms in acute myeloid leukemia integrating biomarkers and treatment sequencing. The figure depicts diagnosis-driven treatment allocation informed by molecular profiling, patient fitness, and risk stratification, integrating targeted therapies, consolidation approaches, and relapse management. It represents an original interpretative synthesis of biomarker-guided AML management developed in alignment with key NCCN and ELN principles. The scheme does not constitute a direct reproduction of any published guideline, figure, or external source. Selected therapeutic pathways, particularly emerging targeted combinations and maintenance strategies, reflect evolving clinical evidence, contemporary translational insights, and investigational treatment approaches currently under active evaluation. All abbreviations employed are defined in the text in the Abbreviations section.

Table 7 summarizes contemporary management of AML, including indications, representative regimens, molecular biomarkers, toxicity considerations, and supporting evidence, while Figure 5 outlines diagnosis-driven therapeutic pathways and treatment algorithms integrating molecular profiling and treatment sequencing.

## 6. Conclusions

The integration of targeted, biology-driven therapies into AML management has reshaped clinical expectations, showing that outcomes can be meaningfully improved when treatment is aligned with disease biology. Experience with midostaurin, venetoclax, and gemtuzumab ozogamicin illustrates that mechanistically distinct strategies—kinase inhibition, apoptosis modulation, and antibody–drug conjugates—can each provide benefit when applied in the right clinical and molecular context. At the same time, these approaches reinforce a central principle: treatment efficacy in AML is not determined by a drug in isolation, but by its interaction with disease biology, therapeutic backbone, and patient-specific factors.

This paradigm places diagnostic precision at the core of clinical decision-making. Rapid molecular and immunophenotypic characterization is now essential to guide therapy selection, moving beyond a simple distinction between intensive and non-intensive treatment. Instead, management increasingly reflects a layered assessment that integrates genetic risk, disease dynamics, and patient fitness, enabling more rational use of targeted agents across different clinical settings.

Despite this progress, key limitations remain. Resistance continues to challenge durable disease control, driven by clonal heterogeneity and adaptive survival mechanisms. Even when responses are achieved, their durability varies substantially, particularly in biologically high-risk disease. In parallel, treatment-related toxicity—most notably prolonged cytopenias and infectious complications—can limit the intensity or continuity of otherwise effective regimens, highlighting the need for improved supportive strategies and treatment optimization.

The growing number of therapeutic options also introduces increasing complexity. Optimal sequencing, combination strategies, and treatment duration are not yet fully defined and are often extrapolated from individual trial designs rather than direct comparative evidence. Addressing these uncertainties will require more integrative clinical trial approaches and the incorporation of dynamic disease monitoring to support adaptive, response-guided treatment decisions.

Equally important is the translation of these advances into routine clinical practice. Access to molecular diagnostics, treatment availability, and multidisciplinary expertise will influence the extent to which precision medicine can be effectively implemented. Ensuring equitable access and sustainable integration of increasingly complex therapies remains a critical challenge.

Overall, the shift toward a diagnosis-driven framework represents a major step forward in AML care, but it remains an evolving process. The therapies discussed in this review demonstrate the potential of targeted approaches, while also underscoring the need for continued refinement in patient selection, treatment integration, and resistance management. Future progress will depend on effectively combining biological insight with clinical strategy, with the ultimate goal of achieving more durable remissions and improving long-term outcomes in this heterogeneous disease.

## Figures and Tables

**Table 1:** TEAEs and management strategies for midostaurin according to [93,94,127]. All abbreviations employed are defined in the text in the Abbreviations section.

TEAE	Frequency/Severity	Timing/Clinical Features	Recommended Management
Cytopenias (neutropenia, anemia, thrombocytopenia)	Very common; frequently grade ≥ 3	During induction and consolidation; often overlaps with chemotherapy-related myelosuppression	Standard supportive care (transfusions, growth factors as indicated); regular blood count monitoring; dose modifications rarely required
Febrile neutropenia/infections	Common; potentially life-threatening	During periods of prolonged neutropenia	Prompt empiric antibiotics; antifungal prophylaxis according to institutional guidelines; close infection surveillance
Pulmonary toxicity (interstitial lung disease, pneumonitis)	Rare but potentially severe	Subacute onset with dyspnea, cough, and hypoxia	Interrupt treatment; perform diagnostic imaging and infectious workup; consider corticosteroids if drug-related
Cardiac events (e.g., heart failure, arrhythmias)	Rare; potentially severe	Variable onset; higher risk in patients with pre-existing cardiovascular disease	Cardiac monitoring in high-risk patients; manage according to cardiology guidelines; consider treatment discontinuation if severe
QT interval prolongation	Uncommon; can be serious	Variable onset; risk increased by concomitant QT-prolonging medications and electrolyte abnormalities	Baseline and periodic ECG monitoring; correction of electrolyte disturbances; avoid interacting drugs; interrupt treatment if clinically significant
Elevated liver enzymes (AST/ALT)	Common; mostly grade 1–2	During treatment cycles; usually reversible	Regular liver function monitoring; dose interruption or reduction for grade ≥ 3 toxicity
Gastrointestinal toxicity (nausea, vomiting, diarrhea)	Very common; mostly grade 1–2	Early onset during induction; may persist during treatment cycles	Prophylactic and therapeutic antiemetics; supportive care; temporary dose interruption for grade ≥ 3 events
Rash/dermatologic reactions	Common; usually mild-to-moderate	Typically occurs early during treatment and is often self-limited	Symptomatic treatment with antihistamines and/or topical corticosteroids; continuation of therapy unless severe
Hypersensitivity/infusion-related reactions	Rare	Usually occurs shortly after administration; may present with fever, rash, or hypotension	Supportive care; antihistamines with or without corticosteroids; discontinue treatment if severe
Other adverse events (metabolic abnormalities, electrolyte disturbances)	Rare; variable severity	Often asymptomatic laboratory abnormalities	Routine laboratory monitoring; correction of abnormalities and supportive management as needed

ALT—alanine aminotransferase; AST—aspartate aminotransferase; ECG—electrocardiogram; TEAE—treatment-emergent adverse event.

**Table 2:** Major pivotal clinical trials and selected emerging studies of midostaurin in AML. All abbreviations employed are defined in the text in the Abbreviations section.

Trial/Study; Trial Period	Population	Cancer Setting	Design	Combination	Key Findings	Inclusion/ Eligibility Criteria
RATIFY/CALGB 10603 (NCT00651261); 2008–2011 [93]	Adults 18–59 years with newly diagnosed FLT3-mutated AML	Frontline intensive therapy	Phase III, randomized, double-blind, placebo-controlled	Midostaurin + daunorubicin/cytarabine induction, high-dose cytarabine consolidation, then maintenance	Significantly improved OS (median 74.7 vs. 25.6 months; HR 0.78, 95% CI 0.63–0.96; *p* = 0.009) and EFS (HR 0.78, 95% CI 0.66–0.93; *p* = 0.002) compared with placebo. CR rates were similar between groups (~59%), establishing midostaurin as standard-of-care therapy for newly diagnosed FLT3-mutated AML.	Newly diagnosed AML; FLT3-ITD or FLT3-TKD mutation; eligible for intensive chemotherapy.
Global phase 3b study/A2408 (NCT03379727); 2018–2021 [98]	Young and older adults with newly diagnosed FLT3-mutated AML	Frontline intensive therapy and maintenance	Phase IIIb, open-label, multicenter, single-arm	Midostaurin + standard induction and consolidation, followed by maintenance	Achieved high CR/CRi rates (~80%) and confirmed the safety and effectiveness of midostaurin in a broader, real-world-like population, including older fit patients. Survival outcomes were consistent with those reported in RATIFY.	Newly diagnosed FLT3-mutated AML; eligible for intensive chemotherapy.
RADIUS (NCT01883362); 2013–2016 [96]	Patients with FLT3-ITD-positive AML in CR1 after allo-HSCT	Post-transplant maintenance	Phase II, randomized, open-label	Standard of care ± midostaurin	Demonstrated feasibility of post-transplant maintenance. Estimated RFS at 18 months was higher with midostaurin (89% vs. 76%), suggesting reduced relapse risk, although differences were not statistically significant and the study was not powered for OS.	FLT3-ITD-positive AML in CR1 post allo-HSCT.
AMLSG 16-10 (NCT01477606); 2012–2017 [94]	Adults 18–70 years with newly diagnosed FLT3-ITD-positive AML	Frontline induction, consolidation, maintenance	Phase II, single-arm	Midostaurin + intensive chemotherapy ± allo-HSCT	High CR/CRi rate (~72%) and favorable long-term outcomes compared with historical controls, supporting improvements in OS and EFS across age groups and transplant strategies.	Newly diagnosed FLT3-ITD AML; intensive-therapy eligible.
Phase Ib Stone et al. study; 2004–2007 [92]	Adults with newly diagnosed AML	Frontline dose-finding	Phase Ib, open-label	Midostaurin + intensive chemotherapy	Demonstrated feasibility of combining midostaurin with intensive chemotherapy, with CR rates comparable to standard induction therapy. Established the recommended dosing schedule used in subsequent studies, including RATIFY.	Newly diagnosed AML; fit for intensive therapy.
SAL MIDOKIT (NCT01830361); 2013–2017 [99]	Patients with t(8;21) AML with KIT and/or FLT3 mutations	Molecularly defined AML subset	Phase II, single-arm	Midostaurin + intensive chemotherapy	Reported encouraging CR rates and favorable survival outcomes in a high-risk molecular subgroup, supporting further investigation of KIT-targeted approaches in CBF AML.	t(8;21) AML with KIT and/or FLT3 mutation.
Phase IIb monotherapy study; 2002–2004 [90]	Relapsed/refractory AML or high-risk MDS	Relapsed/refractory	Phase IIb, open-label	Midostaurin monotherapy	Demonstrated biological and clinical activity, including reductions in peripheral and bone marrow blasts, but responses were generally transient with limited PFS and no durable remissions, highlighting the need for combination strategies.	R/R AML or high-risk MDS; FLT3-mutated or wild-type.

**Table 3:** TEAEs and management strategies for venetoclax according to [56,184,185,186]. All abbreviations employed are defined in the text in the Abbreviations section.

TEAE	Frequency/Severity	Timing/Clinical Features	Recommended Management
Tumor lysis syndrome (TLS)	Uncommon but serious; potentially life-threatening	Typically occurs during cycle 1 dose ramp-up; risk increased in patients with high leukemic burden	TLS prophylaxis (hydration, allopurinol ± rasburicase); stepwise dose ramp-up; inpatient monitoring for high-risk patients
Cytopenias (neutropenia, thrombocytopenia, anemia)	Very common; often grade ≥ 3	Early during cycle 1 and recurrent with subsequent cycles; prolonged neutropenia common	Dose interruptions or delays; G-CSF support; transfusions; bone marrow assessment if cytopenias are prolonged
Febrile neutropenia/infections (bacterial, fungal)	Common; potentially severe	During periods of profound neutropenia, particularly during early treatment cycles	Prompt empiric antibiotics; antifungal prophylaxis (e.g., azoles); infection monitoring; consider dose adjustments
Hemorrhagic events	Uncommon; may be serious	Frequently associated with thrombocytopenia	Platelet transfusions as needed; monitor for signs of bleeding
Drug–drug interactions (CYP3A inhibitors, e.g., azoles)	Clinically significant	Throughout treatment; may substantially increase venetoclax exposure	Venetoclax dose reduction when co-administered with strong or moderate CYP3A inhibitors; careful medication review
Electrolyte disturbances (e.g., hyperkalemia, hypocalcemia, hyperphosphatemia)	Common in TLS context; variable severity	Most frequently observed during treatment initiation	Close electrolyte monitoring; correction of abnormalities; management according to TLS protocols
Hepatic enzyme elevations (AST/ALT)	Common; mostly grade 1–2	During treatment cycles; generally reversible	Monitor liver function tests; interrupt treatment for grade ≥3 toxicity
Gastrointestinal toxicity (nausea, diarrhea, constipation)	Very common; mostly grade 1–2	Early onset; usually manageable	Supportive care; antiemetics and antidiarrheals as needed; maintain adequate hydration
Fatigue/asthenia	Common; usually mild-to-moderate	May occur throughout treatment	Supportive care; evaluate and treat contributing factors such as anemia or infection
Other rare adverse events (e.g., hypersensitivity reactions, cardiac events)	Rare	Variable presentation and timing	Supportive care; treatment discontinuation if severe

AE—adverse event; ALT—alanine aminotransferase; AST—aspartate aminotransferase; CYP3A—cytochrome P450 3A; G-CSF—granulocyte colony-stimulating factor; TEAE—treatment-emergent adverse event; TLS—tumor lysis syndrome.

**Table 4:** Major pivotal clinical trials and selected emerging studies of venetoclax in AML. All abbreviations employed are defined in the text in the Abbreviations section.

Trial/Study; Trial Period	Population	Cancer Setting	Design	Combination	Key Findings	Inclusion/ Eligibility Criteria
VIALE-A (NCT02993523); 2017–2019 [56]	Older/unfit adults with newly diagnosed AML	Frontline non-intensive	Phase III, randomized, double-blind	Venetoclax + azacitidine vs. azacitidine	Significantly improved OS (median 14.7 vs. 9.6 months; HR 0.66, 95% CI 0.52–0.85; *p* < 0.001), CR/CRi rate (66.4% vs. 28.3%), and EFS compared with azacitidine alone. Established venetoclax plus azacitidine as the standard of care for older or unfit patients with newly diagnosed AML.	Newly diagnosed AML; ineligible for intensive chemotherapy; age ≥ 75 years or significant comorbidities.
VIALE-C (NCT03069352); 2017–2019 [187]	Older/unfit adults with newly diagnosed AML	Frontline non-intensive	Phase III, randomized, double-blind	Venetoclax + LDAC vs. LDAC	Improved CR/CRi rate (48% vs. 13%) and demonstrated an OS benefit with extended follow-up (median OS 8.4 vs. 4.1 months; HR 0.70, 95% CI 0.50–0.99). Clinical benefit was less pronounced than in VIALE-A but remained clinically meaningful.	Newly diagnosed AML; ineligible for intensive chemotherapy.
Phase Ib/II DiNardo et al. (NCT02203773); 2014–2017 [163]	Older/unfit patients with newly diagnosed AML	Frontline dose-expansion	Phase Ib/II, open-label	Venetoclax + azacitidine or decitabine	Achieved high CR/CRi rates (67–73%) with rapid median time to response (~1 month) and encouraging OS (median OS up to 17.5 months). These results provided the foundation for the phase III VIALE-A study.	Newly diagnosed AML; age ≥ 65 years; unfit for intensive therapy.
Phase Ib/II Wei et al. (NCT02287233); 2015–2018 [150]	Older/unfit AML patients	Frontline dose-expansion	Phase Ib/II, open-label	Venetoclax + LDAC	Demonstrated a CR/CRi rate of approximately 54% and median OS of 10.1 months, comparing favorably with historical LDAC monotherapy outcomes and supporting further evaluation in VIALE-C.	Newly diagnosed AML; unfit for intensive therapy.
VIALE-A + phase Ib venetoclax + azacitidine study M14-358; 2022 [188]	Newly diagnosed AML patients with IDH1/2 mutations	Frontline non-intensive	Post hoc pooled analysis of phase III and phase Ib studies	Venetoclax + azacitidine	CRc rate 79%, median duration of remission 29.5 months, and median OS 24.5 months in patients with IDH1/2-mutated AML. Demonstrated particularly favorable outcomes with venetoclax–azacitidine in this molecular subgroup.	Newly diagnosed AML; IDH1- or IDH2-mutated disease; ineligible for intensive chemotherapy; enrolled in VIALE-A or phase Ib venetoclax–azacitidine studies.
Relapsed/Refractory AML studies (various); 2016–2023 [146]	Patients with R/R AML	Relapsed/refractory	Phase I/II, predominantly open-label	Venetoclax monotherapy or combinations	Venetoclax monotherapy demonstrated modest activity (ORR ~19–35%; median OS generally <6 months), whereas combination regimens with hypomethylating agents, LDAC, or targeted therapies improved response rates and survival outcomes. These studies also identified mechanisms of resistance, including MCL-1 dependence and clonal evolution.	R/R AML; prior therapies permitted; molecularly heterogeneous populations.
Emerging intensive-combination studies; 2018–present [152,189,190]	Fit patients with newly diagnosed AML	Frontline intensive	Phase Ib/II, ongoing	Venetoclax + intensive chemotherapy (e.g., FLAG-IDA, 7+3)	Venetoclax combined with intensive chemotherapy (e.g., FLAG-IDA or 7+3) achieved high CR/CRi rates (70–90%), substantial MRD-negative remission rates (>60% in several studies), and encouraging EFS and OS outcomes. These approaches remain investigational, with optimal dosing and toxicity management under ongoing evaluation.	Newly diagnosed AML; fit for intensive chemotherapy; generally younger patients.

**Table 5:** TEAEs and management strategies for gemtuzumab ozogamicin according to [64,223,243,244]. All abbreviations employed are defined in the text in the Abbreviations section.

TEAE	Frequency/Severity	Timing/Clinical Features	Recommended Management
VOD/SOS	Uncommon but potentially life-threatening	Risk increased after HSCT, pre-existing liver disease, or short interval between GO administration and transplantation	Assess hepatic risk before treatment; monitor weight, bilirubin, hepatomegaly, and ascites; avoid close sequencing with HSCT when possible; initiate urgent supportive care if suspected
Myelosuppression/prolonged cytopenias	Very common; often grade ≥ 3	During induction or post-treatment recovery; includes neutropenia, thrombocytopenia, and anemia	Frequent CBC monitoring; transfusion support; infection prophylaxis; delay subsequent treatment cycles until recovery
Febrile neutropenia/infections	Common; potentially severe	During neutropenic periods	Prompt empiric broad-spectrum antibiotics; antifungal and antiviral prophylaxis according to institutional practice; close monitoring
Hemorrhagic events	Common; may be serious, particularly in patients with thrombocytopenia	During thrombocytopenic periods; may involve mucosal, gastrointestinal, or intracranial bleeding	Platelet monitoring and transfusion support; management of coagulopathy; interrupt treatment if severe
Hepatotoxicity/elevated AST, ALT, or bilirubin	Common; variable severity	During treatment or early follow-up; often presents as laboratory abnormalities	Baseline and serial liver function monitoring; avoid concomitant hepatotoxic drugs; interrupt or discontinue treatment for severe toxicity
Infusion-related reactions	Common; usually grade 1–2, rarely severe	During or shortly after infusion; manifestations include fever, chills, hypotension, dyspnea, and rash	Premedication with corticosteroids, antihistamines, and antipyretics; close monitoring during infusion; interrupt or discontinue treatment if severe
Gastrointestinal toxicity (nausea, vomiting, diarrhea, mucositis)	Common; mostly grade 1–2	Early during treatment; may overlap with chemotherapy-related toxicity	Antiemetics, hydration, oral care, antidiarrheal therapy, and nutritional support as needed
TLS/metabolic abnormalities	Rare; risk dependent on disease burden	Early after treatment initiation	Hydration, allopurinol or rasburicase when indicated; monitor electrolytes, uric acid, and renal function
Hypersensitivity and other immune-mediated reactions	Rare; may be severe	During or shortly after infusion	Stop infusion; provide supportive care; administer corticosteroids and antihistamines; permanently discontinue treatment in cases of anaphylaxis
Other rare adverse events	Rare; variable severity	Includes cardiac, pulmonary, renal, or severe dermatologic events	Symptom-directed evaluation; specialist consultation; interrupt or discontinue treatment if clinically significant

ALT—alanine aminotransferase; AST—aspartate aminotransferase; CBC—complete blood count; GO—gemtuzumab ozogamicin; HSCT—hematopoietic stem cell transplantation; SOS—sinusoidal obstruction syndrome; TEAE—treatment-emergent adverse event; TLS—tumor lysis syndrome; VOD—veno-occlusive disease.

**Table 6:** Major pivotal clinical trials and selected emerging studies of gemtuzumab ozogamicin in AML. All abbreviations employed are defined in the text in the Abbreviations section.

Trial/Study; Trial Period	Population	Cancer Setting	Design	Combination	Key Findings	Inclusion/ Eligibility Criteria
ALFA-0701 (NCT00927498); 2009–2011 [64,223]	Adults 50–70 years with newly diagnosed de novo AML	Frontline intensive therapy	Phase III, randomized, open-label	GO + daunorubicin/cytarabine vs. chemotherapy alone	Significantly improved EFS (2-year EFS: 40.8% vs. 17.1%; HR 0.58, 95% CI 0.43–0.78; *p* = 0.0003) and RFS (2-year RFS: 50.3% vs. 22.7%; HR 0.52, 95% CI 0.36–0.75; *p* = 0.0003), with a lower cumulative incidence of relapse. No statistically significant improvement in OS was observed. Supported renewed clinical use of fractionated low-dose GO in CD33-positive AML.	Newly diagnosed de novo AML; age 50–70 years; intensive-chemotherapy eligible; APL excluded.
AAML0531 (NCT00372593); 2006–2010 [224]	Children, adolescents, and young adults with newly diagnosed AML	Frontline pediatric AML	Phase III, randomized	Standard chemotherapy ± GO	Reduced relapse risk (RR: 32% vs. 41%; *p* = 0.006) and improved EFS (3-year EFS: 53% vs. 47%; HR 0.83; *p* = 0.04). No significant improvement in OS was observed. Supported incorporation of GO into pediatric AML treatment regimens.	Newly diagnosed AML; pediatric and young adult population; CD33-expressing AML broadly represented.
NCRI AML16; 2006–2012 [226]	Older adults with AML or high-risk MDS	Frontline older/unfit-to-moderately fit AML	Phase III, randomized	Intensive chemotherapy ± GO	mproved OS (3-year OS: 25% vs. 20%; HR 0.87, 95% CI 0.76–1.00; *p* = 0.05), particularly among patients with favorable- or intermediate-risk disease. Findings supported the use of lower-dose GO in selected older adults with AML.	Older adults with AML/high-risk MDS; eligible for trial-defined chemotherapy.
MRC AML15; 2002–2009 [227]	Younger adults with newly diagnosed AML	Frontline intensive therapy	Phase III, randomized	Intensive induction chemotherapy ± GO	No significant improvement in OS was observed in the overall study population; however, favorable-risk patients experienced improved RFS and a reduced cumulative incidence of relapse. These findings contributed to the development of risk-adapted GO treatment strategies.	Newly diagnosed AML; intensive-treatment eligible; cytogenetic and molecular risk assessed.
SWOG S0106 (NCT00085709); 2004–2009 [228]	Adults ≤ 60 years with previously untreated de novo AML	Frontline intensive therapy	Phase III, randomized	Daunorubicin/cytarabine ± GO; post-consolidation GO vs. observation	No significant improvement in CR rate, DFS, or OS was observed. Induction mortality was significantly higher in the GO arm (5.5% vs. 1.4%; *p* = 0.006). These findings highlighted the importance of appropriate dosing, scheduling, and patient selection for GO-based therapy.	Previously untreated de novo non-M3 AML; age ≤ 60 years; intensive-treatment eligible.
MyloFrance-1; 2007–2010 [220]	Adults with first-relapse CD33-positive AML	Relapsed AML	Phase II, open-label	Fractionated single-agent GO	Achieved an ORR of 26%, including a CR/CRp rate of 26%, with a median OS of 8.4 months. Demonstrated clinically meaningful activity of fractionated single-agent GO in first-relapse CD33-positive AML and supported its use in the relapsed/refractory setting.	CD33-positive AML in first relapse; adults; adequate organ function.
GOELAMS AML2006IR; 2007–2013 [231]	Adults with intermediate-risk AML	Frontline intensive therapy	Phase III, randomized	Intensive chemotherapy ± GO	Improved EFS and reduced relapse incidence compared with chemotherapy alone, although no significant OS benefit was observed. Results supported the role of GO in selected patients with intermediate-risk AML, with efficacy influenced by disease biology and treatment context.	Newly diagnosed intermediate-risk AML; intensive chemotherapy eligible.

**Table 7:** Contemporary management of AML: clinically focused overview of current treatment strategies, including indications, representative regimens, biomarkers, toxicity considerations, and supporting evidence. All abbreviations employed are defined in the text in the Abbreviations section.

Modality	Indication/When Used	Example Regimens/Agents	Key Evidence	Biomarkers/Toxicity
Intensive induction chemotherapy	Fit patients with newly diagnosed AML	“7+3” daunorubicin or idarubicin + cytarabine	ELN recommendations; contemporary AML reviews [21,159]	Requires fitness assessment; major toxicities include myelosuppression, infection, mucositis, and organ toxicity.
FLT3-targeted intensive therapy	Newly diagnosed FLT3-mutated AML eligible for intensive chemotherapy	Midostaurin + daunorubicin/cytarabine induction and cytarabine consolidation	RATIFY/CALGB 10603 [93]	FLT3-ITD or FLT3-TKD mutation; monitor cytopenias, gastrointestinal toxicity, rash, QT prolongation, and drug interactions.
Low-intensity venetoclax-based therapy	Older or medically unfit patients with newly diagnosed AML	Venetoclax + azacitidine; venetoclax + decitabine; venetoclax + LDAC	VIALE-A; VIALE-C [56,187]	Useful in intensive-ineligible AML; toxicities include prolonged cytopenias, infections, tumor lysis syndrome, and CYP3A-mediated interactions.
CD33-directed antibody–drug conjugate therapy	CD33-positive AML, particularly favorable/intermediate-risk or selected newly diagnosed disease	GO + daunorubicin/cytarabine; fractionated GO schedules	ALFA-0701; AAML0531; AML15 meta-evidence [64,223,224]	CD33 expression; strongest benefit often seen in favorable/intermediate-risk AML; monitor hepatotoxicity, thrombocytopenia, infusion reactions, and VOD/SOS.
CPX-351 liposomal chemotherapy	Therapy-related AML or AML with myelodysplasia-related changes	Liposomal daunorubicin/cytarabine	Phase III CPX-351 trial [297]	Used in secondary-type AML; toxicities include prolonged cytopenias, infections, bleeding, and cardiotoxicity monitoring.
Targeted therapy for IDH-mutated AML	Newly diagnosed unfit or relapsed/refractory IDH1- or IDH2-mutated AML	Ivosidenib; olutasidenib; enasidenib; combinations with azacitidine	AGILE and IDH inhibitor studies [298,299]	IDH1/IDH2 mutations; monitor differentiation syndrome, QT prolongation, leukocytosis, and hepatic abnormalities.
Post-remission consolidation	Patients achieving CR after induction	High-dose cytarabine; risk-adapted consolidation; transplant-directed strategies	ELN recommendations [21]	Guided by ELN risk, MRD, cytogenetics, and mutations; toxicities include cytopenias, neurotoxicity, infection, and organ toxicity.
Allogeneic HSCT	Intermediate/adverse-risk AML, MRD-positive disease, or selected relapsed disease	Allogeneic hematopoietic stem cell transplantation	ELN recommendations and transplant studies [21,159]	Risk-adapted by cytogenetics, mutations, MRD, donor availability, and fitness; toxicities include graft-versus-host disease, infection, relapse, and non-relapse mortality.
Maintenance therapy	Patients in remission not proceeding to transplant or after intensive therapy; selected post-HSCT settings	Oral azacitidine; FLT3 inhibitor maintenance in FLT3-mutated AML	QUAZAR AML-001; FLT3 maintenance studies [96,300]	MRD and mutation status may guide selection; monitor cytopenias, gastrointestinal toxicity, infections, and drug-specific adverse events.
Relapsed/refractory AML therapy	Disease recurrence or refractory AML after frontline treatment	Venetoclax-based salvage; FLT3 inhibitors; IDH inhibitors; GO in selected CD33-positive relapse; clinical trials	ELN recommendations; pivotal targeted-agent studies [21,159]	Requires repeat molecular testing; toxicity depends on regimen but commonly includes cytopenias, infections, hepatic toxicity, and differentiation syndrome with IDH inhibitors.
Supportive and palliative care	All AML phases; especially frail patients or those not eligible for disease-directed therapy	Transfusions, antimicrobials, growth factors, hydroxyurea, symptom-directed care	ASH/ELN guidance [21,301]	Focus on infection prevention, bleeding control, transfusion support, quality of life, and treatment goals.

## Data Availability

No new data were created or analyzed in this study. Data sharing is not applicable to this article.

## Abbreviations

The following abbreviations are used in this manuscript:

7+3	cytarabine (7 days) plus anthracycline (3 days)
ADCs	antibody–drug conjugates
ADCC	antibody-dependent cellular cytotoxicity
AE	adverse event
AKT	protein kinase B
ALFA-0701	Acute Leukemia French Association 0701 trial
allo-HSCT	allogeneic hematopoietic stem cell transplantation
ALT	alanine aminotransferase
AML	acute myeloid leukemia
APL	acute promyelocytic leukemia
ASH	American Society of Hematology
AST	aspartate aminotransferase
BAX	Bcl-2-associated X protein
BCL-2	B-cell lymphoma 2
BCL-X_L_	B-cell lymphoma-extra large
BIM	Bcl-2-interacting mediator of cell death
BM	bone marrow
CAR-T	chimeric antigen receptor T-cell therapy
CBC	complete blood count
CBF	core-binding factor
CD33	cluster of differentiation 33
CD34+	cluster of differentiation 34–positive cells
CD123	cluster of differentiation 123
CI	confidence interval
CLAD	cladribine-based regimen
CR	complete remission
CR1	first complete remission
CRc	composite complete remission
CRi	complete remission with incomplete hematologic recovery
CRp	complete remission with incomplete platelet recovery
CYP3A	cytochrome P450 3A
DFS	disease-free survival
DNA	deoxyribonucleic acid
ECG	electrocardiogram
ECHO	echocardiography
ECOG	Eastern Cooperative Oncology Group
EFS	event-free survival
ELN	European LeukemiaNet
ERK	extracellular signal-regulated kinase
Fab	fragment antigen-binding
Fc	fragment crystallizable
FDA	U.S. Food and Drug Administration
FLAG-IDA	fludarabine, cytarabine, granulocyte colony-stimulating factor, idarubicin
FLT3	Fms-like tyrosine kinase 3
FLT3-ITD	Fms-like tyrosine kinase 3 internal tandem duplication
FLT3-TKD	Fms-like tyrosine kinase 3 tyrosine kinase domain mutation
G-CSF	granulocyte colony-stimulating factor
GO	gemtuzumab ozogamicin
HMA(s)	hypomethylating agent(s)
HR	hazard ratio
HSCT	hematopoietic stem cell transplantation
IDH(1/2)	isocitrate dehydrogenase (1/2)
ITD	internal tandem duplication
KIT	KIT proto-oncogene receptor tyrosine kinase
LDAC	low-dose cytarabine
LFTs	liver function tests
LSCs	leukemia stem cells
MCL-1	myeloid cell leukemia 1
MDS	myelodysplastic syndrome
MEK	Mitogen-activated protein kinase kinase
MRD	measurable residual disease
NCCN	National Comprehensive Cancer Network
NCT	ClinicalTrials.gov identifier
NGS	next-generation sequencing
NPM1	nucleophosmin 1
ORR	overall response rate
OS	overall survival
OXPHOS	oxidative phosphorylation
PFS	progression-free survival
PI3K	phosphoinositide 3-kinase
pSTAT5	phosphorylated signal transducer and activator of transcription 5
QALY(s)	quality-adjusted life year(s)
RADIUS	midostaurin maintenance trial
RAF	Rapidly accelerated fibrosarcoma kinase
RAS	rat sarcoma viral oncogene homolog
RATIFY	CALGB 10603 trial
RFS	relapse-free survival
RFT	renal function tests
R/R	relapsed/refractory
RR	relapse risk
SOS	sinusoidal obstruction syndrome
STAT5	signal transducer and activator of transcription 5
t(8;21)	translocation between chromosomes 8 and 21
TEAE	treatment-emergent adverse event
TKD	tyrosine kinase domain
TKI	tyrosine kinase inhibitor
TLS	tumor lysis syndrome
TP53	tumor protein p53
VEN	venetoclax
VIALE-A	venetoclax plus azacitidine trial
VIALE-C	venetoclax plus low-dose cytarabine trial
VOD	veno-occlusive disease
Y694(Tyr694)	Tyrosine residue at position 694
